# The Relationship Between Neuromuscular Block Depth and Airway Retroglossal Area: A Prospective, Nonrandomized, Observational Clinical Trial

**DOI:** 10.3390/jcm14124374

**Published:** 2025-06-19

**Authors:** László Asztalos, Mena Boktor, Miklós Kukuly, Dorka Sólyom, Adrienn Pongrácz, Sorin J. Brull, Béla Fülesdi

**Affiliations:** 1 Department of Anesthesiology and Intensive Care, Faculty of Medicine, University of Debrecen, Nagyerdei krt. 98., H-4032 Debrecen, Hungary; asztalos.laszlo@med.unideb.hu (L.A.); mena.boktor45@gmail.com (M.B.);; 2 Department of Pulmonology, Faculty of Medicine, University of Debrecen, Nagyerdei krt. 98., H-4032 Debrecen, Hungary; kukuly.miklos@med.unideb.hu; 3 Department of Anesthesiology and Perioperative Medicine, Mayo Clinic College of Medicine, San Pablo Rd., Jacksonville, FL 4500, USA; sjbrull@me.com

**Keywords:** train-of-four ratio, neuromuscular recovery, airway, retroglossal area, tracheal extubation, postoperative pulmonary complications

## Abstract

**Background**: Tracheal intubation and mechanical ventilation are facilitated by neuromuscular blocking agents. We investigated the effectiveness of subjective clinical evaluation of neuromuscular function on retroglossal area size, since it determines spontaneous ventilation adequacy following tracheal extubation. Secondarily, we correlated changes in retroglossal area and depth of neuromuscular block assessed during both respiratory phases using quantitative neuromuscular monitoring. **Methods**: Once mechanical ventilation was no longer needed, antagonists were used to reverse the neuromuscular block in 21 consenting patients; adequacy of reversal was assessed subjectively by delivering a sequence of four rapid (2 Hz) electrical stimuli (train-of-four, TOF) to a peripheral nerve and assessing attainment of four equal muscle contractions (TOF ratio = 1.0), signifying normal neuromuscular function. Retroglossal area during both inhalation and exhalation were measured pharyngoscopically at various phases of neuromuscular recovery, including at baseline after anesthesia induction but before neuromuscular block onset and at recovery before tracheal extubation; area changes were correlated with depth of quantitatively measured neuromuscular block. **Results**: Clinicians’ subjective evaluation of readiness for tracheal extubation failed to identify significant residual block in most patients who required rescue antagonism. Markedly decreased retroglossal areas (inhalation: 39.5% of baseline; exhalation: 20.1% of baseline) were present at extubation, and 11 out of 21 (52.4%) patients needed rescue antagonism. In contrast, in patients with neuromuscular recovery to the currently recommended threshold determined quantitatively (TOF ratio > 0.90), retroglossal areas were only 80% recovered but returned to near baseline values when the TOF ratio ≥ 0.95. **Conclusions**: Quantitative monitoring should guide the timing of tracheal extubation. Current definitions of the minimal threshold for adequate neuromuscular recovery (TOF ratio > 0.90) after mechanical ventilation in postoperative patients should be re-evaluated. A TOF ratio > 0.95 better correlates with return to normal (baseline) retroglossal area during both inhalation and exhalation.

## 1. Introduction

Perioperative monitoring of the depth of neuromuscular block and adequacy of neuromuscular recovery after mechanical ventilation are patient care issues that have recently been addressed by the publication of national [1,2] and international guidelines [3,4]. Although these guidelines made strong recommendations for the use of quantitative neuromuscular monitoring to exclude residual paralysis and ensure adequate recovery, defined as a train-of-four (TOF) ratio of at least 0.90, the regular use of quantitative neuromuscular monitors has yet to become part of everyday practice.

The incidence of residual neuromuscular block is highly underestimated by clinicians. A survey that included 2636 responses from European and American anesthesiologists found the estimated frequency of clinically significant residual neuromuscular block in routine practice to be less than 1% [5]. However, the actual incidence exceeds 60% even in academic hospitals and 80% in non-academic hospitals [6]. In fact, most clinicians believed that neither conventional peripheral nerve stimulators nor quantitative TOF monitors should be part of minimum monitoring standards [5]. The low rate of quantitative monitor use has been attributed by most trainees and consultants to low confidence in these monitors [7]. Most clinicians, regardless of practice location, still rely primarily on clinical (subjective) signs to decide on the use of reversal agents and the time of extubation, even though the sensitivity and specificity of these signs are known to be very low [8]. This practice is difficult to change because even among clinicians who are interested in the topic, a significant lack of knowledge about neuromuscular block monitoring and overconfidence in their own knowledge have been reported [9].

The purpose of this study was to investigate residual neuromuscular block in routine clinical care and to also determine to what extent this residual block affects the patency of the retroglossal area, the most sensitive area to obstruction after tracheal extubation [10]. Our secondary goal was to study the correlation between the change in the retroglossal area and the depth of neuromuscular block assessed during both respiratory phases using quantitative neuromuscular monitoring.

## 2. Materials and Methods

### 2.1. Ethics Approval and Registration

Declarations: This prospective, nonrandomized, observational clinical trial was performed at the University of Debrecen, Department of Anaesthesiology and Intensive Care, between April 2024 and January 2025 and followed the World Medical Association’s Declaration of Helsinki. The study protocol was approved by the local institutional (DE RKEB/IKEB:625-2022) and national (OGYEI/3962-11-/2023) medical ethics committees. All patients provided written informed consent to participate in the study. The reporting of results followed the Strengthening the Reporting of Observational Studies in Epidemiology (STROBE) guidelines [11]. The trial was registered at: ClinicalTrials.gov (NCT06544980) (https://clinicaltrials.gov/study/NCT06544980?term=NCT06544980&rank=1, registration date: 5 August 2024).

We chose board-certified anesthesiologists from our department whose routine practice did not involve the use of quantitative neuromuscular monitoring during general anesthesia (“clinical anesthesiologists”). They were asked to perform general anesthetics according to their daily routine. Thus, in all surgical cases, the choice of muscle relaxant, the time of intubation, administration of intraoperative top-up doses, and the time of extubation were all left to the discretion of the clinical anesthesiologist based on his/her routine practice. For determining the most appropriate time for tracheal extubation, the clinical anesthesiologist relied on patients’ clinical signs such as head lift, hand grip, and adequacy of tidal volume during spontaneous ventilation, as per their routine practice. Although the clinical anesthesiologist was guided by the routine subjective evaluation of the clinical signs, an independent research anesthesiologist performed quantitative neuromuscular measurements intraoperatively using an electromyography monitor. The clinical anesthesiologist was blinded to the results of intraoperative quantitative monitoring but was given these results after tracheal extubation to ensure full neuromuscular recovery and patient safety.

### 2.2. Pharyngoscopic Recordings

**Figure 1:**
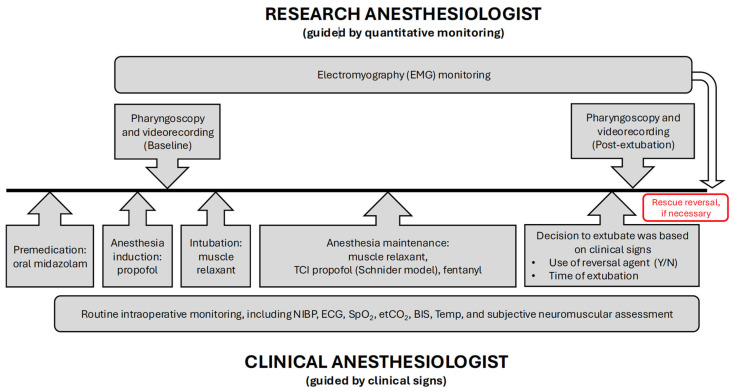
Flowchart—diagrammatic representation of the sequence of intraoperative events from preoperative premedication until tracheal extubation and end of surgery. EMG: electromyography; TCI: target-controlled infusion; NIBP: non-invasive blood pressure; ECG: electrocardiography; SpO_2_: peripheral oxygen saturation; etCO_2_: end-tidal carbon dioxide; BIS: bispectral index; Temp: temperature.

To compare the upper airway cross-sectional area before tracheal intubation (baseline) and after extubation, two pharyngoscopic examinations were performed (Figure 1). The first (baseline) pharyngoscopy was performed under propofol anesthesia but before the administration of opioids and muscle relaxants to ensure that the patient maintained spontaneous ventilation and avoid the effects of opioids on pharyngeal muscle function. The second pharyngoscopic recording was obtained after tracheal extubation (Figure 1).

**Figure 2:**
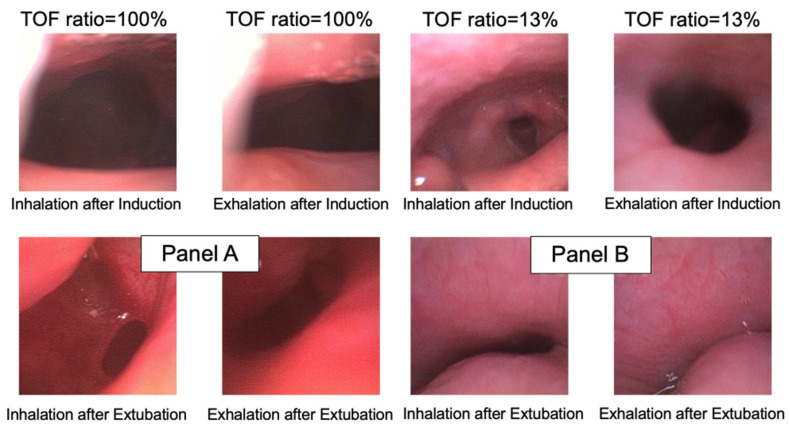
Demonstrative cases of retroglossal area during inhalation and exhalation after induction (baseline) and after tracheal extubation. Panel (**A**) is from a patient with a train-of-four ratio = 1.0 (100%); panel (**B**) is from a patient with a train-of-four ratio = 0.13 (13%). TOF: train-of-four.

During pharyngoscopy, a continuous chin lift (Esmarch–Heiberg maneuver) was performed, while an Ambu^®^ aScope™ 4 RhinoLaryngo Slim endoscope (Ambu A/S, Ballerup, Denmark) was advanced nasally to visualize the vocal cords. The vocal fold was used as a landmark. From this position, the camera was slowly retracted to locate the narrowest part of the pharynx, which corresponded to the retroglossal region (Figure 2). The depth of nasal insertion was marked on the endoscope to ensure identical depth of insertion during subsequent recordings. During both pharyngoscopic examinations, video images were recorded, both during the inhalation and exhalation respiratory phases, for subsequent off-line analysis.

### 2.3. Anesthetic Management

According to the institutional protocol and as part of a balanced anesthetic technique, patients received premedication with 7.5 mg midazolam orally. After anesthesia induction with intravenous (IV) propofol, a target-controlled propofol infusion was used according to the Schnider model [12]. Routine intraoperative monitoring included electrocardiography, pulse oximetry, non-invasive blood pressure, core body temperature, and end-expiratory carbon dioxide measurement. The research anesthesiologist used a TetraGraph^®^ (Senzime AB, Uppsala, Sweden) electromyograph, which stimulates a peripheral nerve and detects and records the compound muscle action potentials using single-use electrodes (TetraSens, Senzime). The stimulating electrodes were placed on the volar surface of the forearm 2–3 cm proximal to the wrist crease for ulnar nerve stimulation, and the recording electrodes were placed on the adductor pollicis muscle (active electrode) and distally on the thumb (reference electrode) as recommended [13]. Nerve stimulation was started after the induction of anesthesia but before the administration of neuromuscular blocking agents (NMBAs). The neuromuscular monitor performs an autocalibration and determines the supramaximal current needed to ensure consistent muscle contraction and establishes the amplitude (in mV) of the baseline compound muscle action potential. The monitor used the TOF stimulation pattern every 20 sec to record the TOF ratio, TOF count, and post-tetanic count (PTC) data.

During anesthesia induction, the target propofol plasma dose was set at 4–6 µg/mL such that the targeted depth of anesthesia monitor’s bispectral (BIS) index (BIS Monitoring System, Medtronic, Minneapolis, MN, USA) was maintained between 60 and 70%. A baseline pharyngoscopy was performed before opioids and muscle relaxants were administered to record the upper airway cross-sectional area. This process ensured that patients had reached a sufficient hypnotic state while maintaining spontaneous ventilation that allowed assessment of the upper airway during both the inspiratory and expiratory phases. Once the recording was complete, the patient was given intravenous fentanyl and the NMBA chosen by the clinical anesthesiologist, as per usual routine, followed by tracheal intubation. Subsequently, the anesthetic was deepened for the duration of the operation, to reach a target BIS index of 40–60. The target concentration of propofol infusion was then changed to 2.5–4 µg/mL for maintenance of general anesthesia.

At the end of surgery, the clinical anesthesiologist decided on the timing of tracheal extubation and communicated with the research anesthesiologist, who recorded the actual neuromuscular monitor parameters. The clinical anesthesiologist was blinded to the actual quantitative neuromuscular monitoring results (TOF ratio) as per usual clinical routine, unless they revealed significant residual neuromuscular block that might have affected patient safety. A second pharyngoscopy was performed immediately after tracheal extubation by the research anesthesiologist. After decreasing the dose of propofol, pharyngoscopic measurements were repeated when an identical depth of anesthesia (bispectral index: 60–70) was reached as during the first pharyngoscopic measurement. To ensure that the pharyngoscopy recording was performed at the same depth of the upper airway as the preoperative baseline measurement, a continuous chin lift was used and then the pharyngoscope was introduced nasally up to the marker placed during the baseline measurement.

### 2.4. Safety Measures

Following the post-extubation pharyngoscopy, a neuromuscular reversal agent (sugammadex for antagonism of aminosteroidal agents or neostigmine for antagonism of benzylisoquinolinium agents) was recommended to the clinical anesthesiologist if the quantitatively obtained post-extubation TOF ratio was below 0.9 (90%).

### 2.5. Measurement of Upper Airway Area

Using software available online, Image J (Rasband WS, U.S. National Institutes of Health, Bethesda, MD, USA; https://imagej.net/ij/, accessed 15 February 2025), the size of the airway area was traced, and the program automatically allocated the number of pixels that corresponded to the area of interest. After anesthesia induction (pre-intubation phase), the largest upper airway area was registered and traced, independent of the respiratory phase. Three measurements of this area were performed by two independent anesthesiologists who were not involved in the clinical part of the study. The results of the 3 measurements were averaged, and these values were used for further statistical analysis. During the post-extubation phase, a set of three measurements were performed during both the inhalation and exhalation phases, and the resulting inhalation and exhalation areas were averaged and used for statistical analysis.

### 2.6. Statistical Analysis

Sample size calculation: In a previous investigation [14], the authors used a magnetic resonance imaging technique to describe the minimal retroglossal diameter after total neuromuscular recovery of 20.2 ± 5.2 mm, and a 20% decrease in this diameter at a TOF ratio = 0.8. We hypothesized that in the present study, the retroglossal cross-sectional area during inhalation would decrease by 30% in patients with residual neuromuscular block (defined as a TOF ratio < 0.90) of any severity. Using an alpha of 0.05 and a power of 90%, we calculated that 8 patients per group would be necessary to test our hypothesis. In a previous study, we found that in our routine practice, the incidence of residual neuromuscular block without monitoring during spontaneous recovery approximates 45% [15]. We thus planned to include a total of 21 patients to account for any potential patient drop-out or failures in monitoring.

The number of pixels (area) of the regions of interest was recorded during both the inhalation and exhalation phases. After a normality test, medians and interquartile ranges were calculated for the different areas, which were then compared by Kruskal–Wallis One-Way Analysis of Variance on Ranks. As a second step, we calculated the percent change in the upper airway cross-sectional area during the post-extubation inspiratory and expiratory phases by dividing the post-extubation values by the postinduction (baseline) values and multiplying by 100. These percent changes were then compared using the Wilcoxon signed-rank test. Statistical differences were considered significant if *p* < 0.05.

## 3. Results

Twenty-one patients (nine females and twelve males) were enrolled in the study. The pertinent factors related to the surgical intervention are summarized in Table 1.

Table 2 describes the intraoperative neuromuscular management of the patients.

Different types of neuromuscular blocking agents were used, including amino-steroidal (rocuronium and pipecuronium) and benzylisoquinolinium (atracurium and cisatracurium) compounds. Tracheal extubation based on traditional subjective (qualitative) means resulted in 12 of 21 (57.1%) patients being considered adequately recovered, and 7 (33.3%) received no neuromuscular block antagonism from the clinical anesthesiologist. Nine of the nineteen (47.4%) patients who had received amino-steroid NMBAs required rescue sugammadex administration after extubation, and both (100%) patients who received benzylisoquinolinium NMBAs required neostigmine rescue reversal. There were no other complications in any of the 21 patients.

Among the patients in whom the timing of tracheal extubation was decided by clinical criteria, all but one (patient 20, TOF count = 1) had a TOF count = 4 at extubation. Interestingly, in those patients in whom the clinical anesthesiologists decided to use reversal agents (*n* = 9) after tracheal extubation, only three patients met the criteria for minimum recovery based on quantitative neuromuscular monitoring. Rescue reversal medication was necessary due to insufficient return of neuromuscular function at the time of tracheal extubation in 11 (52.4%) patients (10 received sugammadex and 1 received neostigmine).

### 3.1. Retroglossal Area and Depth of Block

We compared the area of the upper airway (retroglossal area) after induction of anesthesia but before tracheal intubation at end-exhalation and end-inhalation. After extubation, areas were grouped according to the degree of neuromuscular recovery (TOF ratios) and were compared (Table 3). Retroglossal areas in the TOF ratio < 0.90 patient group (*n* = 11; 52.4%) were lower during both the inspiratory and expiratory phases compared to the baseline measurements. In contrast, retroglossal areas in the TOF ratio ≥ 0.90 group (*n* = 10) were only marginally lower compared to baseline values in both respiratory phases.

### 3.2. Retroglossal Area Change and Extubation

**Figure 3:**
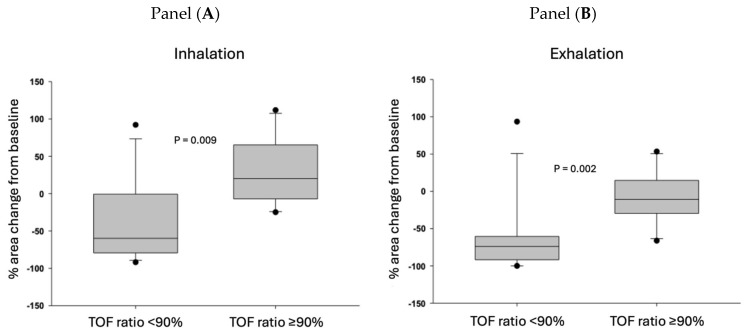
Percent change in the upper airway compared to the baseline upper airway cross-sectional areas during inhalation (Panel (**A**)) and exhalation (Panel (**B**)). Patients are grouped based on the train-of-four ratio measured immediately after extubation. The threshold of neuromuscular recovery is defined as a train-of-four ratio ≥ 0.90. Medians and 25–75% interquartile ranges are presented. The horizontal middle line represents the median, the grey boxes represent the 25–75% confidence intervals, the “error bars” represent minimum and maximum values, and the dots represent outliers.

We calculated the relative upper airway area changes by dividing the areas at inhalation and exhalation by the baseline area. Figure 3 shows the relative change in upper airway area during inspiratory and expiratory phases after extubation as a function of the degree of neuromuscular recovery (TOF ratio after tracheal extubation). The data demonstrate that if the TOF ratio < 0.90 at extubation, the upper airway (retroglossal area) was significantly narrowed compared to the post-induction (baseline) retroglossal area in both the inspiratory and expiratory phases: the median value of the upper airway area was only 20–30% of the baseline area. In contrast, in the patients whose tracheas were extubated when the TOF ratio ≥ 0.90, the median area of the preserved upper airway (retroglossal area) was above 80% of baseline.

**Figure 4:**
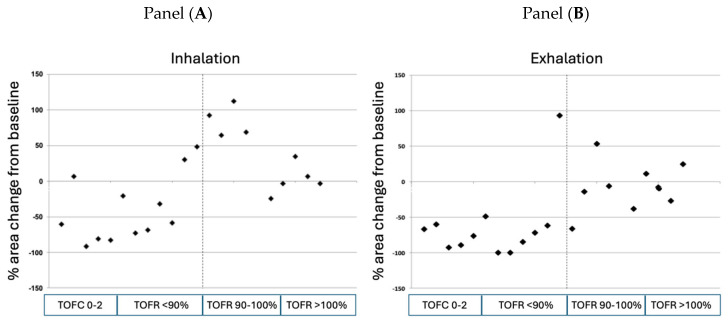
Individual measurements of retroglossal area during inhalation (Panel (**A**)) and exhalation (Panel (**B**)) immediately after tracheal extubation as a function of the degree of neuromuscular recovery. The dotted line represents the threshold of neuromuscular recovery, defined as a train-of-four ratio > 0.90. TOFC: train-of-four count; TOFR: train-of-four ratio.

As a final step, we plotted the individually measured TOF ratios at extubation against the percent changes in retroglossal area measured during inhalation and exhalation. Figure 4 demonstrates that the narrowing of the upper airway area has a close relationship with the TOF ratios both during inhalation and exhalation. The line of TOF ratio = 0.90 (defining minimal acceptable neuromuscular recovery) differentiates clusters of preserved vs. significantly narrowed upper airway areas both during inhalation and exhalation: out of the twelve patients exhibiting a TOF ratio < 0.90 after extubation, seven (58.3%) patients had an upper airway narrowing that exceeded 50% during the inspiratory phase. During exhalation, 10 of the 11 (91%) patients were found to experience significant residual neuromuscular block (TOF ratio < 0.90). In the ten patients with TOF ratio ≥ 0.90, two (20%) patients showed moderate narrowing of the upper airway area; in the remaining eight patients, the upper airway area was either comparable with the postinduction values or was larger after tracheal extubation than the baseline area.

## 4. Discussion

Although the most recent guidelines [3,4] recommend against the use of clinical signs (response to verbal commands such as opening the eyes, squeezing the hand, or lifting the head) or subjective evaluation of “four full twitches” in response to TOF peripheral nerve stimulation because they are notoriously poor indicators of adequate neuromuscular recovery, the use of quantitative neuromuscular monitoring has not yet become part of routine daily practice. Consequently, the incidence of residual neuromuscular block (TOF ratio < 0.90) has been reported to exceed 80% in non-academic institutions and roughly 60% in academic hospitals in the United States [6].

In the present study, in an academic hospital in Europe, we investigated those anesthesiologists whose routine practice did not include quantitative neuromuscular monitoring but instead made their tracheal extubation decisions based on the patients’ clinical signs. We recorded the upper airway cross-sectional area at baseline and after the administration of NMBA, and the depth of neuromuscular block in parallel, allowing us to correlate the change in retroglossal area as a function of the depth of neuromuscular block. We found that clinical assessment of the readiness for tracheal extubation failed to identify significant residual neuromuscular block in 57.1% of our patients. It should be emphasized that in 9 of the 21 patients, the anesthesiologist considered reversal necessary (5 used neostigmine and 4 used sugammadex) during the clinical decision-making, but the use of a rescue reversal agent (based on quantitative monitoring) was still necessary in 11 cases; 4 of these patients had already received neostigmine reversal. From a patient safety perspective, these four patients inappropriately received neostigmine antagonism from a depth of block at which neostigmine is neither effective nor recommended [3]. Our findings are consistent with the previous literature documenting the inadequacy of clinical signs in guiding appropriate management of neuromuscular block [16] and the high rate of residual block in routine care that is not guided by quantitative monitoring [6].

The strong correlation between the depth of neuromuscular block (TOF ratio) and airway patency is illustrated in Figure 3; in cases with TOFR > 0.90, all data points are clustered around the 0 line, indicating no or minimal change from the initial retroglossal area. In patients in whom a TOF ratio > 0.90 was measured at the time of extubation, the retroglossal airway area had returned to the initial, pre-intubation value both in the inspiratory and expiratory phases. However, if the TOF ratio < 0.90, the median value of the retroglossal airway area decreased by more than 50% compared to baseline in both phases of the respiratory cycle.

We also noted that the relative retroglossal area change was less in the inspiratory phase than in the expiratory phase. Similar to the present study, a decreased retroglossal and retropharyngeal upper airway volume has been documented in patients with partial neuromuscular block [14]. The authors also documented that retroglossal upper airway diameter was especially decreased during forced inhalation: 74 ± 18% at a TOF ratio of 0.5, and 81 ± 18% at a TOF ratio of 0.8 [14]. Using electromyographic recordings of the genioglossus muscle, the authors also demonstrated that even low degrees of neuromuscular block were responsible for a narrowed upper airway. Because of inadequate genioglossus muscle function, the negative pressure generated by the respiratory muscles on the airway cannot be counteracted with an appropriate opening force of the upper airway dilator muscles, and the flaccid upper airway structures are narrowed; this may cause a decreased forced vital capacity, decreased peak expiratory and inspiratory flows [17], and a markedly decreased closing pressure of the upper airway [10].

Although a TOF ratio > 0.9 is a widely accepted threshold for safe tracheal extubation after the use of NMBAs, in a further sub-analysis, we compared the retroglossal areas of patients in whom the TOF ratio was 0.85–0.94 with patients in whom the TOF ratio was >0.95. The results are summarized in Table 4.

A significant difference could be demonstrated in the retroglossal airway area between the two groups, both in the inspiratory and expiratory phases. It is important to underscore that the retroglossal area returned close to pre-induction values in patients in whom the TOF ratio was >0.95 at extubation. Thus, our data show that the upper airway cross-sectional area remains decreased from baseline even when the current threshold of recovery recommended by the latest clinical guidelines (e.g., TOF ratio ≥ 0.90) has been reached [3,4]. This is consistent with previous observations [17,18] that reported upper airway obstruction even though the TOF ratio had recovered to values between 0.9 and 1.0 (and above the currently accepted threshold of TOF > 0.9). Our data also suggest that for the upper airway cross-sectional area to return to near-baseline values, the threshold of neuromuscular recovery of ≥ 0.9 is necessary, but ≥ 0.95 is probably safer. This is also consistent with work performed 6 decades ago, which demonstrated that when the neuromuscular response to a single stimulus had returned to normal, 75–80% of the postsynaptic receptors can still be blocked [19,20].

Our study has certain limitations. Although the number of cases (*n* = 21) is limited, we reproduced a common postoperative clinical scenario. The differences between the degree of retroglossal area narrowing in patients with a TOF ratio < 0.9 and > 0.9 are statistically and, more importantly, clinically significant, even in this relatively small number of patients. We recognize that the number of cases may not provide sufficient evidence to formulate a definitive proposal for changing the threshold of neuromuscular recovery to a TOF ratio >0.95. However, our findings are consistent with, and supportive of, previous reports [17,18,21].

There might also be criticism regarding the consistency of the method of endoscopic measurement of the retroglossal area. However, every precaution was taken to ensure that the endoscope’s position after induction of anesthesia and after extubation did not change; in both cases, the jaw thrust maneuver was used, and the depth of insertion of the endoscope was marked at the alar rim to ensure a consistent depth of insertion. In the images obtained by video recording of both inspiratory and expiratory phases, the images showing the narrowest airway diameter were fixed, and the corresponding number of pixels was recorded. We could not numerically determine the size of the retroglossal area using this method, but our primary goal was to determine the relative area changes after induction and after extubation, and to correlate these changes with the depth of neuromuscular block.

We documented the consequences of mismanagement of neuromuscular block and antagonism that is based on the still-common use of subjective evaluation and clinical signs of residual paralysis. Our most important finding is that when the TOF ratio is still below the currently accepted TOF ratio of 0.90, the retroglossal upper airway is significantly narrowed. In young, otherwise healthy patients, this airway narrowing can be compensated for by increased work of breathing, without significant clinical consequences. However, in certain groups of patients (elderly patients and obese patients with sleep apnea), the narrowing of the airway could pose a significant respiratory risk.

Another important finding is the identification of the inadequate, but still present, practice of using neuromuscular antagonists without guidance from objective neuromuscular monitoring. Neostigmine administered without quantitative monitoring when the TOF count is 3, for instance, obtained from a peripheral nerve stimulator, may create a false sense of security that extubation can be performed reliably 5–10 min after neostigmine administration. Such is not the case, however. Sadly, the consistent use of quantitative neuromuscular monitors has not become part of everyday practice, despite international recommendations and statements [1,2,3,4]. Changing misconceptions [9,13,22], regular training programs on neuromuscular monitoring and reversal, and changing intraoperative patient safety requirements can (and must) lead to a reduction in complications of residual neuromuscular block. Our findings strongly support the use of quantitative monitoring to guide the timing of tracheal extubation in critically ill intubated patients and underscore the inadequacy of neuromuscular management based on clinical signs and subjective evaluation. Current definitions of the minimal threshold for adequate and safe neuromuscular recovery after intubation and mechanical ventilation in critically ill patients should be re-evaluated.

## Figures and Tables

**Table 1:** Patient characteristics and surgical data. ASA PS: American Society of Anesthesiologists Physical Status; F: female; M: male; BMI: body mass index.

Patient	ASA PS	Sex (F/M)	Age (Years)	BMI	Type of Surgery	Surgery Duration (min)	Anesthesia Duration (min)
1	II	F	61	20	Laparoscopic cholecystectomy	35	65
2	II	F	37	28	Laparoscopic cholecystectomy	30	55
3	I	F	32	26	Laparoscopic cholecystectomy	40	60
4	III	M	46	18	Ileostomy	60	85
5	II	M	45	26	Herniotomy	40	75
6	II	M	32	28	Laparoscopic colon resection	110	120
7	II	M	62	24	Laparoscopic herniorrhaphy	60	85
8	II	F	55	26	Laparoscopic cholecystectomy	40	95
9	II	F	39	31	Laparoscopic cholecystectomy	70	85
10	II	F	63	25	Laparoscopic cholecystectomy	30	50
11	II	F	48	23	Laparoscopic cholecystectomy	30	55
12	II	M	63	28	Ileum resection	190	210
13	II	M	63	27	Laparoscopic herniorrhaphy	30	40
14	I	M	19	19	Laparoscopic cholecystectomy	30	45
15	II	F	50	26	Laparoscopic cholecystectomy	30	55
16	II	M	26	28	Subtotal colectomy	300	320
17	II	F	62	24	Laparoscopic rectum resection	195	250
18	II	M	57	27	Preperitoneal inguinal hernia repair	55	95
19	II	M	32	21	Ileum resection	160	180
20	II	M	43	24	Laparoscopic hemicolectomy	100	110
21	II	M	52	25	Thyroidectomy	70	110

**Table 2:** Intraoperative neuromuscular management. NMBA: neuromuscular blocking agent; SGX: sugammadex; NEO: neostigmine; TOF: train-of-four; ROC: rocuronium; PIP: pipecuronium; ATR: atracurium; CIS: cisatracurium. Numbering of the patients is identical to Table 1.

	NMBA Type	NMBA Intubation Dose (mg)	NMBA Top-Up Dose (mg)	Reversal (Y/N)	Time from Last NMBA Dose Until Reversal (min)	Sgx Dose (mg)	Neo Dose (mg)	Time from Reversal to Extubation (min)	TOF Count at Extubation (n)	TOF Ratio at Extubation (%)	Rescue SGX (Y/N)	Rescue NEO (Y/N)
1	ROC	30	10	No	No reversal	0	0	-	4	49	Yes	No
2	ROC	40	0	Yes	50	50	0	5	4	90	No	No
3	ROC	40	0	No	No reversal	0	0	-	4	10	Yes	No
4	PIP	4	0	Yes	75	0	2	15	0	0	Yes	No
5	ROC	50	0	No	No reversal	0	0	-	4	86	No	No
6	PIP	8	2	Yes	15	100	0	10	4	102	No	No
7	ROC	40	0	No	No reversal	0	0	-	4	96	No	No
8	ROC	40	20	No	No reversal	0	0	-	4	11	Yes	No
9	ROC	50	40	Yes	35	0	2	10	1	0	Yes	No
10	ROC	40	0	No	No reversal	0	0	-	4	13	Yes	No
11	ATR	30	0	Yes	30	0	2	15	4	11	No	Yes
12	PIP	8	1	Yes	40	100	0	10	4	108	No	No
13	PIP	8	0	Yes	30	0	2	15	1	0	Yes	No
14	CIS	10	0	Yes	35	0	2	10	2	0	No	Yes
15	ROC	35	0	Yes	40	100	0	5	4	100	No	No
16	ROC	50	60	No	No reversal	0	0	-	4	42	Yes	No
17	ROC	50	0	No	No reversal	0	0	-	4	96	No	No
18	ROC	50	0	No	No reversal	0	0	-	4	100	No	No
19	ROC	40	10	No	No reversal	0	0	-	4	100	No	No
20	PIP	8	0	No	No reversal	0	0	-	1	0	Yes	No
21	ROC	40	0	No	No reversal	0	0	-	4	106	No	No

**Table 3:** Retroglossal baseline area (number of pixels) in patients with train-of-four ratio < 90% and train-of-four ratio ≥ 90% at extubation. Depending on the results of the normality tests, means ± standard deviations or medians (25–75% interquartile ranges) are shown. TOF: train-of-four.

	TOF Ratio < 90% (*n* = 11)	TOF Ratio ≥ 90% (*n* = 10)
	**Inhalation**	
Retroglossal baseline area after induction (pixels)	277,371 ± 73,171	213,904 ± 51,447
Retroglossal area after extubation	109,444 (60,823–246,313)	265,989 ± 84,826
Recovery compared with baseline	39.5%	124.3%
	**Exhalation**	
Retroglossal baseline area after induction (pixels)	166,854 ± 19,359	142,665 ± 48,741
Retroglossal area after extubation	33,498 (17,778–51,179)	191,743 ± 70,306
Recovery compared with baseline	20.1%	134.4%

**Table 4:** Percent change in the retroglossal airway area after extubation (baseline) during inhalation and exhalation in patients with a train-of-four ratio = 0.85–0.94 and in those with a train-of-four ratio > 0.95. Medians and 25–75% interquartile ranges are shown in parentheses. TOF: train-of-four.

	TOF Ratio = 0.85–0.94 *n* = 13	TOF Ratio > 0.95 *n* = 8	*p* Value
Inhalation (% change)	−59.0 (−75.4 to 12.1)	5.972 (−7.1 to 71.1)	0.023
Exhalation (% change)	−71.7 (−90.0 to 57.2)	−14.1 (−29.6 to 14.7)	0.008

## Data Availability

Data will be made available by the corresponding author upon reasonable request.
